# Genetic evidence for GLP1R agonists in non-ischaemic heart failure

**DOI:** 10.1093/eschf/xvag077

**Published:** 2026-03-14

**Authors:** Nhu Ngoc Le, Dipender Gill, Sandosh Padmanabhan

**Affiliations:** BHF Cardiovascular Research Centre, School of Cardiovascular & Metabolic Health, University of Glasgow, 126 University Place, Glasgow G12 8TA, UK; Department of Epidemiology and Biostatistics, School of Public Health, Imperial College London, London W2 1PG, UK; BHF Cardiovascular Research Centre, School of Cardiovascular & Metabolic Health, University of Glasgow, 126 University Place, Glasgow G12 8TA, UK

**Keywords:** GLP1RA, Non-ischaemic heart failure, HFpEF, BMI, Glycemia, Drug-target Mendelian randomization

## Abstract

**Background:**

Glucagon-like peptide-1 receptor (GLP1R) agonists reduce cardiovascular events in patients with obesity and diabetes, and recent trials demonstrate symptomatic and functional benefits in heart failure with preserved ejection fraction (HFpEF). Whether these benefits reflect glycaemic control, weight reduction, or additional mechanisms remains uncertain.

**Methods:**

We performed drug-target Mendelian randomization (MR) using genetic variants in the GLP1R-locus to proxy GLP1R activation. MR estimates for GLP1R agonism were scaled to per 0.1 log-odds lower liability to type 2 diabetes (T2DM) and compared with general type 2 diabetes (T2DM) or body mass index (BMI) lowering effects. Summary statistics were obtained from the largest available GWAS of HF, non-ischaemic HF (ni-HF), ni-HFpEF, ni-HFrEF, and atrial fibrillation (AF). Primary inverse-variance weighted analysis was adjusted for multiple testing and validated via weighted median and MR-Egger sensitivity analyses.

**Results:**

Genetically proxied GLP1R activation was associated with lower risk of overall HF [OR 0.96 (95% CI 0.95–0.98), *P* & .0002], ni-HF [0.94 (0.91–0.96), *P* & .0001], and ni-HFpEF [0.82 (0.74–0.90), *P* & .0001] per 0.1 log-odds lower T2DM liability. The GLP1R-proxied effects were greater than those expected from glycaemic lowering alone and were of comparable magnitude to those from BMI reduction. Associations for ni-HFrEF were directionally adverse but not significant. AF showed a nominal, exploratory association consistent with BMI lowering and driven by a single *cis*-BMI variant.

**Conclusions:**

This study provides genetic support for a protective association between GLP1R activation and HF, particularly ni-HFpEF, with effects beyond glycaemia. The magnitude and subtype specificity are consistent with contemporary trial evidence and support further evaluation of GLP1R agonism in cardio-metabolic HFpEF.

## Background

Heart failure (HF) is a major cause of morbidity and mortality, with limited therapies targeting HF with preserved ejection fraction (HFpEF), a major subtype that is frequently non-ischaemic. Glucagon-like peptide-1 receptor (GLP1R) agonists reduce cardiovascular events in obesity and diabetes, and recent trials demonstrate symptomatic and functional benefits in HFpEF.^1^ Whether these effects arise predominantly through glycaemic control, weight reduction, or additional mechanisms remains uncertain. Mendelian randomization (MR) provides an opportunity to test causal effects of genetically proxied GLP1R activation on HF outcomes. Prior MR studies reported protective associations but used scaling that limited clinical interpretability,^2^ highlighting the need for clarification.

## Aims

We aimed to evaluate the causal relationship between GLP1R activation and HF, including aetiological subtypes (non-ischaemic HF, HFpEF, and HFrEF), using drug-target MR. By scaling genetic estimates to clinically interpretable units and benchmarking against glycaemia and BMI instruments, we sought to bridge genetic target validation with clinical trial outcomes, provide HF subtype specificity, and clarify mechanistic pathways.

## Methods

We constructed two sets of genetic instruments to proxy GLP1R agonism through distinct metabolic pathways.^3^ The first set comprised single-nucleotide polymorphisms (SNPs) within 100 kb *GLP1R* (GRCh38 chr6: 39 048 781–39 091 303), in low linkage disequilibrium (*r*^2^ < 0.1), associated with T2DM liability at genome-wide significance (*P* < 5 × 10^−8^) in a GWAS meta-analysis including 242 283 cases and 1 327 447 controls of European ancestry.^4^ To ensure biological relevance, variants were directionally concordant and nominally associated with HbA1c (*P* < .05).^5^ These instruments were scaled to per 0.1 log-odds lower T2DM liability, a clinically interpretable unit that avoids inflation from per 1 mmol/mol HbA1c reporting.

The second set includes SNPs associated with BMI in a GWAS meta-analysis of around 806 834 individuals of European ancestry.^6,7^ Although these summary statistics were released alongside studies of fat distribution, the instrument used here reflects BMI rather than waist-hip ratio. Because no GLP1R-proximal variants reached *P* < 5 × 10^−8^, we applied a less stringent threshold (*P* < 1 × 10^−6^), yielding a single *cis* variant. This locus-specific BMI instrument was therefore considered exploratory. For comparison, we also generated genome-wide instruments for overall T2DM liability and BMI using standard clumping (r^2^ < 0.001) to provide robust benchmarks.

Outcome summary statistics were obtained from the largest publicly available GWAS, predominantly of European ancestry, with sample sizes of 1 946 349 for HF and 1 030 836 for AF.^8,9^ Analyses covered overall HF, non-ischaemic HF (ni-HF), non-ischaemic HF with preserved ejection fraction (ni-HFpEF), non-ischaemic HF with reduced ejection fraction (ni-HFrEF), and atrial fibrillation (AF). The overall HF phenotype was defined as any diagnosis of HF, ascertained through physician adjudication, hospital record review, or diagnostic coding.^9^ All contributing studies had ethical approval and participant consent.

MR analyses were performed using inverse variance weighted (IVW) regression as the primary MR method, complemented by weighted median and MR Egger.^10,11^ Multiple testing was controlled with Benjamini–Hochberg FDR. Associations were considered significant at IVW *P* < .05 and *q* < 0.05, with nominal associations defined as *P* < .05 with *q* ≥ 0.05. To compare MR estimates for GLP1R agonism with general T2DM- or BMI-lowering effects, we scaled all estimates to a standard reference (0.1-logOR T2DM or 1-SD BMI decrease). Differences between drug-target and general effects were assessed using the propagation-of-error method to determine if the resulting contrast significantly differed from zero.^12^

All analyses used the TwoSampleMR package in R 4.2.3.

## Results

**Figure 1:**
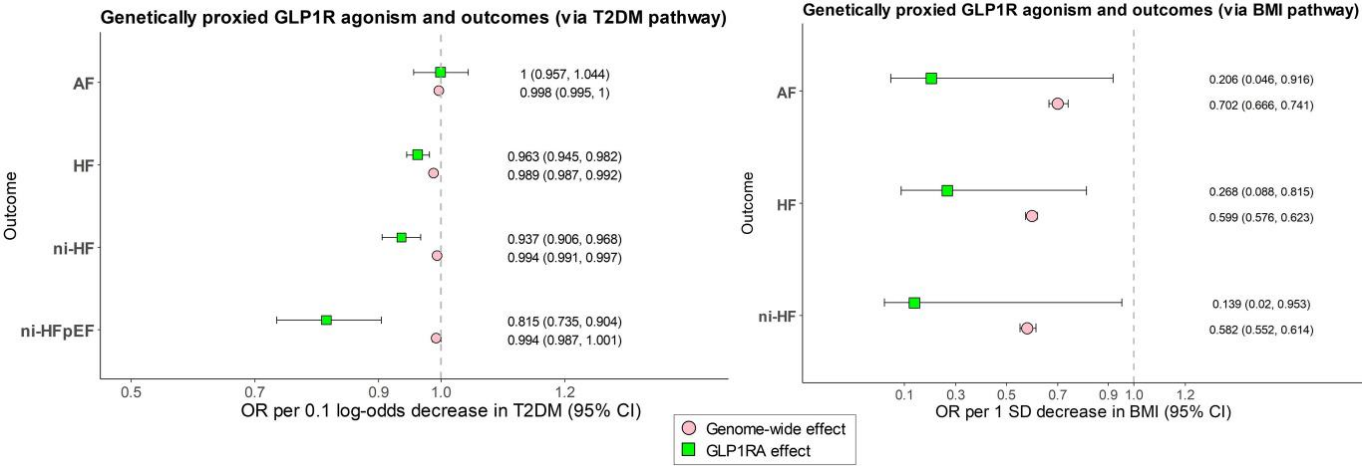
MR estimates for genetically proxied GLP1R agonism via BMI and T2DM pathway, compared with general BMI and T2DM reduction. The left forest plot presents (i) MR estimates for genetically proxied GLP1R agonism via the T2DM pathway across outcomes and (ii) corresponding MR estimates for general genetically proxied T2DM lowering across outcomes. The right forest plot presents (i) MR estimates for genetically proxied GLP1R agonism via the BMI pathway across outcomes and (ii) corresponding MR estimates for general genetically proxied BMI lowering across outcomes. Differences between GLP1R-proxied and general T2DM-/BMI-lowering effects were assessed using the propagation-of-error method (Results)

Using instruments scaled to per 0.1 log-odds lower T2DM liability, genetically proxied GLP1R activation was associated with lower HF risk. Significant associations were observed for overall HF (OR 0.96; 95% CI 0.95–0.98; *q* < 0.05) and ni-HF [OR 0.94 (0.91–0.96); *q* < 0.05]. The largest effect was evident for ni-HFpEF [OR 0.82 (0.74–0.90); *q* < 0.05] (*Figure 1*). MR sensitivity analyses produced estimates of similar direction and magnitude, with no evidence of horizontal pleiotropy. Associations for ni-HFrEF were directionally adverse (OR > 1) but not statistically significant. Given the risk of collider bias from excluding antecedent ischaemic, valvular, and congenital disease in the ni-HFrEF phenotype,^9^ we performed MVMR adjusting for CAD liability; the GLP1R—ni-HFrEF association was still directionally adverse but non-significant (*P* > .05). No significant associations were observed between GLP1R agonism and AF using T2DM instruments.

The GLP1R-locus BMI instrument comprised a single SNP identified using a relaxed significance threshold. This variant yielded nominal associations with overall HF and AF but lacked statistical power, precluding robust sensitivity analyses. These findings are therefore considered exploratory and presented in *Figure 1*.

To contextualize the findings, we compared effect sizes of drug-target perturbation with those derived from genome-wide T2DM liability and BMI instruments (*Figure 1*). GLP1R-proxied effects exceeded those expected from glycaemic lowering alone [overall HF, beta difference & 0.27 (0.07–0.46), *P* & .007; ni-HF, 0.6 (0.26–0.94), *P* & .0004; ni-HFpEF, 1.98 (0.94–3.01), *P* & .0001]. The GLP1R-proxied effects were of comparable magnitude to those from BMI reduction [HF, 0.81 (−0.31–1.92), *P* & .16; ni-HF, 1.43 (−0.49–3.35), *P* & .15]. For AF, the magnitude of association was similar to that observed for BMI lowering, although this finding should be interpreted cautiously, given the exploratory nature of the analysis.

Collectively, the results indicate that GLP1R activation reduces risk of HF—particularly ni-HFpEF—with effect sizes that are clinically plausible and consistent with recent GLP1R trials in obesity and HFpEF. The absence of significant benefit in ni-HFrEF and the modest, BMI-consistent association with AF highlights heterogeneity across outcomes.

## Conclusion

This genetic analysis provides support for a protective association between GLP1R activation and heart failure, with the strongest effects observed for non-ischaemic HFpEF when estimates are scaled to clinically interpretable units. Expressing effects per 0.1 log-odds lower liability to type 2 diabetes yields magnitudes consistent with contemporary GLP1R agonist trials in obesity and HFpEF.^1,13^ Our results suggest that the benefits of GLP1R activation extend beyond glycaemic lowering and are comparable to those expected from BMI reduction, implying an integrated cardio-metabolic profile that includes weight loss, improved haemodynamic, and anti-inflammatory mechanisms.^14^ Associations for non-ischaemic HFrEF were not statistically significant, consistent with heterogeneous and largely neutral trial evidence in this phenotype.^15^

Key limitations include limited generalizability beyond European ancestry populations, the lifelong nature of genetic proxies compared with pharmacological treatment, and the exploratory nature of BMI-mediated analyses based on single-variant instrumentation.

Overall, these findings complement emerging clinical trial data and support further evaluation of GLP1R agonism in cardio-metabolic HFpEF, including in high-risk populations without diabetes.

## Supplementary Material

xvag077_Supplementary_Data

## Data Availability

No new data were generated for this manuscript. This study analysed existing data from publicly available sources, which are cited in the manuscript. The results of the analyses are reported in the main text.
